# Expression of Resuscitation-Promoting Factor C Stimulates the Growth of *Mycobacterium bovis* BCG and Delays DevR Regulon Activation in Hypoxia

**DOI:** 10.1155/ijm/2139933

**Published:** 2025-02-17

**Authors:** Luz Virginia Reyes-González, Olga N. Hernández de la Cruz, Mauricio Castañón-Arreola

**Affiliations:** Genomic Sciences Program, Autonomous University of Mexico City, Mexico City, Mexico

## Abstract

Latent tuberculosis is characterized by the presence of dormant, nonreplicating (DNR) bacilli for years without causing clinical signs and symptoms, remaining as a major reservoir for active tuberculosis. The mechanism through which *M. tuberculosis* transits from DNR to active bacilli remains unclear. However, resuscitation-promoting factors (Rpfs) could participate in the reactivation. Using recombinant *M. bovis* BCG that expresses *rpfC* (*M. bovis* BCG-pMV261::*rpfC*), we evaluated the role of RpfC in the growth of bacilli and the expression of 11 hypoxia-regulated genes in comparison with *M. bovis* BCG-pMV261. The strains were grown in normoxic (21% O_2_), hypoxic (5% O_2_), and anoxic (< 0.1% O_2_) conditions. In normoxic culture, *M. bovis* BCG-pMV261::*rpfC* displays a lower expression of *sigB* and *fdxA.* In anoxic culture, we did not observe drastic changes in the gene expression, except for those involved in electron transport during anaerobic respiration (*pdxA*, *pfkB,* and *nark2*), whose expression was significantly lower in *M. bovis* BCG-pMV261. When the strains were cultured in hypoxia, significantly higher culturability was observed in *M. bovis* BCG-pMV261::*rpfC* compared to *M. bovis* BCG-pMV261. This response was accompanied by a higher *sigB* and *sigE* expression. In both strains, we observed a higher *dosT, devR, fdxA,* and *fpkB* expression in response to hypoxia. Interestingly, except for *fdxA,* the expression of these genes was lower in *M. bovis* BCG-pMV261::*rpfC*. The protein profiles of *M. bovis* BCG-pMV261::*rpfC* reflected the maintenance of an active replicative state (similar to that of the strain grown in normoxic conditions). In anoxic cultures, no significant changes were observed in the expression of hypoxia-response genes. These findings suggest that *rpfC* may have a significant physiological role in inducing the growth of *M. bovis* BCG-pMV261::*rpfC,* which results in the delayed activation of genes related to the transition to anaerobic metabolism.

## 1. Introduction

According to the World Health Organization, *Mycobacterium tuberculosis* infects one-fourth of the world's population. *M. tuberculosis* has evolved as a specialized intracellular pathogen that survives inside diverse host cells, mainly macrophages, causing an asymptomatic subclinical disease or latent infection [1]. Latent tuberculosis is the most common manifestation of the *M. tuberculosis* infection, wherein the bacteria can survive dormant (a nonreplicative stage with a slowdown of division and metabolism) in hypoxic conditions in granulomas for several years [2, 3]. *M. tuberculosis* usually infects phagocytes and resides in granulomas; however, the bacteria may also be found in adipocytes, endothelial cells, and cells of the liver, spleen, and kidney without histological evidence of infection [4, 5]. Under laboratory conditions, the bacteria are cultured with a partial oxygen pressure (PO_2_) of 160 mmHg; however, in vivo, the PO_2_ decreases considerably to 40 mmHg in the capillaries, while in the interstitial liquid, the PO_2_ ranges from 10 to 20 mmHg and is 10 mmHg at the level of the cell membrane (just over 1% of O_2_), while in the tuberculosis, granulomas are in the range of 1.61 ± 0.31 mmHg (∼0.2%) [6–8]. In vivo, *M. tuberculosis* survives in a hypoxic environment where the PO_2_ varies widely according to cell type [9]. Hypoxia activates the *M. tuberculosis dosR-dosS-dosT* system (also known as DevR regulon), which induces the transcription of approximately 50 coregulated genes that may mediate their adaptation to hypoxic and anaerobic conditions and their transition to dormant nonreplicating stage being able to persist maintain oxygen consumption in low nano-oxia environments (0–02 mm Hg) [10, 11].

The biochemical changes that allow *M. tuberculosis* to reactivate are still unknown. However, several studies indicate that it is associated with the upregulation of genes linked to transcription and translation, the synthesis of cell wall compounds, replication, and cell division, including the genes encoding the five resuscitation-promoting factors (Rpfs) that stimulate the growth of *M. tuberculosis* [12–15]. In the *M. tuberculosis* genome, five genes are homologous to the Rpfs of *Micrococcus luteus*, called *rpfA-E* [16, 17]. The addition of recombinant Rpfs to low cell density cultures of *M. luteus* and *M. smegmatis* reduces their latency phases, a phenomenon that has also been observed in cultures of *M. tuberculosis* stimulated with RpfA, RpfC, RpfD, and RpfE proteins [12, 13, 18].

Supplementing starving cultures of *M. tuberculosis* with recombinant Rpfs increases the viability and growth of bacteria despite prolonged incubation times [18]. Rpfs were the first proteins described as inducers of dormant bacteria reactivation [14, 17], and they also promote the survival of mycobacteria in infected mice [19]. Studies have shown that adding Rpfs to sputum samples improves culture positivity by over 80% and increases the recovery of *M. tuberculosis* colony-forming units (CFU) [20]. Mutation of the active site of the Rpfs has revealed that the peptidoglycan hydrolytic activity of these proteins is indispensable for the resuscitation and persistence of *M. tuberculosis* [14, 19]. There is no evidence that the five Rpfs participate together, suggesting that they might be activated through independent mechanisms in *M. tuberculosis*. However, their expression could influence the expression of the others, as suggested by the rapid transcriptional activation of *rpfB* and *rpfC,* which is followed by the consecutive transcriptional activation of the other *rpfs* when the bacilli are exposed to oxygen [21, 22].

How the bacilli are maintained during the dormant state and reactivated is not yet clearly known; however, it is suspected that Rpfs are paramount for the reactivation of *M. tuberculosis*. Experiments with strains defective in *rpf* genes confirm their importance for *M. tuberculosis* growth and their contribution to the reactivation of noncultivable mycobacteria [12, 21]. Only multiple deletions of *rpfs* (*rpfA*-*rpfC-rpfD* or *rpfA-rpfB-rpfC*) result in defective resuscitation in vitro, less virulence, and reduced proliferation in infected mice [21]. Although the Rpfs activity is not fully understood, the expression of these proteins is essential for the reactivation and growth of bacilli, and a relationship has been observed between enzymatic activity and the ability to reactivate mycobacteria. The expression of Rpf proteins varies in different experimental models; in the hypoxia model, except for *rpfC*, a significant decrease in the relative expression of *rpfA, rpfB, rpfD,* and *rpfE* is observed [23, 24]. In latently infected mice, *rpfC* expression remains constant [25], and in the dormant bacilli exposed to oxygen or fresh medium, the transcriptional activation of *rpfB* and *rpfC* occurs very soon [22]. On the other hand, the presence of antibodies against RpfC in the sera of healthy individuals is indirect evidence of the expression of *rpfC* during latency or subclinical disease [26].

For this reason, in this study, we evaluate the effect of *rpfC* overexpression on the growth and the expression of 11 hypoxia-response genes in recombinant *M. bovis* BCG that overexpresses *rpfC* (BCG-pMV261::*rpfC*) in response to different oxygen tension conditions that mimic that found in tissue and necrotic granulomas. Under normoxic culture conditions, no significant differences were observed in the growth of *M. bovis* BCG-pMV261::*rpfC*. However, this strain showed higher cultivability and sustained growth in hypoxic conditions compared to *M. bovis* BCG-pMV261. In hypoxic culture, the overexpression of *rpfC* was accompanied by lower expression of genes related to anaerobic metabolism and the DevR regulon *dosT* and *devR genes*.

## 2. Material and Methods

### 2.1. pMV261::*rpfC Construction*

The full-length *rpf*C gene (Rv1884c, Gene ID: 885,759) was amplified by PCR from genomic DNA of *M. tuberculosis* H37Rv using a high-fidelity DNA Taq polymerase (Thermo Fisher Scientific, Invitrogen, Waltham, MA, U.S.A.) and primers rpfCF-*BamHI* (5′-TCGGGATCCGTGCATCCTTTGCCG-3′) and rpfCR-*HindIII* (5′-CGAAAGCTTTCAGCGCGGAATACT-3′). The PCR products were digested with the appropriate restriction enzymes, purified, and cloned in-frame in the expression vector pMV261 under the control of the hsp60 promoter to obtain the pMV261::*rpfC*. The construction was analyzed and corroborated by the digestion of the vector with BamHI and HindIII restriction enzymes and PCR (using the primers designed for cloning). The gene sequence was confirmed on both strands.

### 2.2. *M. bovis* Culture and Transformation

*M. bovis* BCG Pasteur strain was used to overexpress RpfC protein (*M. bovis* BCG-pMV261::*rpfC*). The strain was cultured in Middlebrook 7H9 broth medium supplemented with 10% albumin–dextrose–catalase (ADC) and 0.05% Tween-80 (both from Becton–Dickinson), and grown at 37°C under constant shaking (200 rpm) until the midlog phase (OD_λ600nm_ = 0.6). Then, the culture was harvested by centrifugation at 5000 rpm, and the bacterial pellet was used to prepare electrocompetent cells. Electrocompetent cells were prepared as previously described [27]. The bacterial pellet was resuspended in a 10% glycerol solution equivalent to the culture media. The bacteria were then centrifuged at 5000 rpm for 10 min at 4°C, and the bacterial pellet was resuspended in 1/2 volume of 10% glycerol. This procedure was repeated until the bacterial suspension was 50X concentrated. The cells were frozen and stored at −70°C until use.

For transformation, *M. bovis* BCG electrocompetent cells were gently mixed with 1 μg of pMV261 or pMV261::*rpfC* plasmids, incubated for 30 min on ice, and then transfected using the Gene Pulser Xcell (Bio-Rad Laboratories, Hercules, CA, USA) applying two pulses of 2.5 kV. Immediately, the cells were gently resuspended in fresh medium and incubated at 37°C with constant agitation for 2 h. Finally, the transfected cells were plated on Middlebrook 7H10 agar media supplemented with oleic acid–albumin–dextrose catalase (OADC) and 30 μg/mL kanamycin and incubated for 20 days. The recovered colonies of *M. bovis* BCG-pMV261 (used as control) and *M. bovis* BCG-pMV261::*rpfC* were cultured in Middlebrook 7H9 broth medium supplemented with ADC and 30 μg/mL kanamycin. The recovered colonies were analyzed to confirm the presence of the plasmids (for *M. bovis* BCG-pMV261) and the presence of *rpfC* in the pMV261::*rpfC* vector by PCR. For growth curve comparison, the strains were cultured in Middlebrook 7H9 broth medium supplemented with ADC, 30 μg/mL kanamycin, and 0.05% Tween-80 for 21 days at 37°C under constant shaking (200 rpm). The OD_λ600nm_ was measured at 2-day intervals using a spectrophotometer.

### 2.3. Cell Viability Determination

To determine the effect of anoxia and hypoxia on the cell viability of *M. bovis* BCG-pMV261::*rpfC*, the same number of CFUs of *M. bovis* BCG-pMV261 and *M. bovis* BCG-pMV261::*rpfC* were plated in triplicate on Middlebrook 7H10 agar plates supplemented with OADC and 30 μg/mL kanamycin for 3 days at 37°C. Then, one plate of each strain was incubated for 3 days at 37°C under anoxia (O_2_ < 0.1%, 7%–15% CO_2_) using AnaeroGen bags (Anaerobic atmosphere generation bags, Thermo Scientific, USA) or 5 days in hypoxia (5% O_2_, 7%–15% CO_2_) using an anaerobic jar. Furthermore, the plates were incubated for 12 days at 37°C under normoxic conditions. The third plate was incubated for 20 days at 37°C under normoxic conditions. Finally, CFUs were counted, and the percentage of cell growth was determined relative to plates of *M. bovis* BCG-pMV261 and *M. bovis* BCG-pMV261::*rpfC* incubated in normoxic conditions. Three biological replicates per condition were done.

### 2.4. Gene Expression Assays

*M. bovis* BCG-pMV261 and *M. bovis* BCG-pMV261::*rpfC* were grown in Middlebrook 7H10 agar plates supplemented with OADC and 30 μg/mL kanamycin for 7 days at 37°C. Then, as described above, one plate of each strain was incubated for 3 days in anoxia or 5 days in hypoxia. Other plates were inoculated and cultured in parallel for the same time at 37°C under normoxic conditions. At the end of the incubation, the cultures were harvested and stored in TRIzol (Gibco BRL, Grand Island, NY, USA) at −70°C until use.

### 2.5. RNA Extraction

Total RNA from *M. bovis* BCG-pMV261 and *M. bovis* BCG-pMV261::*rpfC* was extracted with the TRizol method (Gibco BRL, GRAND Island, NY, USA) following the manufacturer recommendations. Strains were resuspended in TRIzol and transferred to a sterile tube containing 0.1-mm glass beads equivalent to 500 μL. The bacilli were mechanically lysed using a Mini-Beadbeater-1 blender (Bio Spec Products Inc., USA), applying five cycles of 30 s at Speed Level 6, maintaining the tube on ice for 1 min between each cycle. Then, the samples were centrifuged for 5 min at 2000 rpm, and 500 μL of the supernatant was transferred to a new tube. For RNA purification, an equal volume of absolute ethanol was added to each sample, mixed by inversion to extract the RNA using the Direct-Zol RNA MiniPrep kit (Zymo Research, Irvine, CA, USA) according to the manufacturer's protocol. Finally, the RNA was eluted with nuclease-free water and quantified by spectrometry 260/280 nm. The quality and integrity of the RNA were assessed by 1% agarose gel electrophoresis.

### 2.6. qPCR Assay

We evaluated by quantitative real-time PCR (qPCR) the relative expression of 11 genes whose expression increases during the transition of replicative bacilli to the nonreplicative state or the reactivation. The genes are related to hypoxic stress response (*dosT*, *devR, katG*, and *ahpC*), nitrate reduction (*narX* and *narK2*), transcriptional regulation (*sigB*, *sigE,* and *rpoA*), the transition from aerobic to anaerobic metabolism (*fdxA* and *fpkB*), and reactivation (*rpfC*). The *gapdh* expression was used as a reference for data normalization and quantitative gene expression analyses. Briefly, 400 ng of total RNA was reverse transcribed using the SuperScript II (Thermo Fisher Scientific) according to the manufacturer's recommendations. Gene expression was determined using qPCR Luminaris HiGreen High ROX master mix 2X (Thermo Fisher Scientific, USA) on an ABI 7300 Real-Time PCR System (Applied Biosystems, Foster, CA, USA). For each reaction, 10 ng of cDNA and 7.4 pM of specific primers for each gene were used (Table 1). The relative gene expression was calculated using the 2^−ΔΔCT^ method. The experiments were conducted in duplicate for the anoxic cultures and triplicate for the hypoxic cultures.

### 2.7. 2D-PAGE Analysis

*M. bovis* BCG-pMV261 and *M. bovis* BCG-pMV261::*rpfC* were cultured under normoxic and hypoxic conditions as described above. Cells were harvested, and the pellet was resuspended and washed with sterile water and resuspended in 20 mM Tris-HCl, pH 8.0, containing protease inhibitors cocktail (cOmplete-EDTA Free, Roche Mannheim, Germany) and PSMF (2 mM). The cell suspension was transferred to a cryotube containing 0.1-mm glass beads. Bacteria were lysed using the Mini-bead Beater blender (Daigger, USA), applying four cycles of 30 s at Speed 42 and keeping the tube on ice for one minute between cycles. The homogenates were centrifuged at 14,000 rpm for 10 min, and the supernatant was recovered and sterilized by filtration with 0.22-μm membranes (Merck-Millipore, Burlington, MA, USA). Proteins were quantified by the Lowry method, and their integrity was verified on 12.5% SDS-PAGE gels. Proteins were visualized by staining with Coomassie R250 blue.

Proteins of *M. bovis* BCG-pMV261::*rpfC* and *M. bovis* BCG-pMV261::*rpfC* were separated in IPG strips with an immobilized pH gradient from 4 to 7 (GE Healthcare ImmobilineTM DryStrip). Strips were rehydrated with 100 μg of protein extracts suspended in rehydration buffer (9 M urea, 4% CHAPS, 70 mM DTT, a trace amount of bromophenol blue, and 2% ampholytes pH 4–7). After 16 h of passive rehydration, the isoelectric focusing (IEF) was performed in five steps: (1) 500 V × 30 min, (2) 1000 V × 30 min, (3) 1500 V × 30 min, (4) 2000 V × 30 min, and (5) 5000 V until reaching 50,000 Vh. The strips were reduced and alkylated by subsequent incubation in equilibrium buffer (6 M urea, 30% glycerol, 50 mM Tris pH 8.8, 2% SDS, and 0.002% bromophenol blue) plus 15 mg/mL DTT or 37.5 mg/mL IAA, respectively. Then, proteins were separated on 12.5% SDS-PAGE gels and visualized by staining with Sypro Ruby Protein Gel Stain (Thermo Fisher Scientific, Waltham, MA, USA) following the manufacturer's recommendations. The gels were analyzed using the PDQuest 8.0 software (Bio-Rad, Hercules, CA, USA).

### 2.8. Statistical Analysis

Bacterial growth rates and data from qPCR assays were analyzed using two-way ANOVA test for multiple comparisons, with Bonferroni as a posttest. Statistical analyses were conducted using Prism software (Dotmatics, Bishop's Stortford, UK). Differences with *p* < 0.05 were considered significant.

## 3. Results

**Figure 1:**
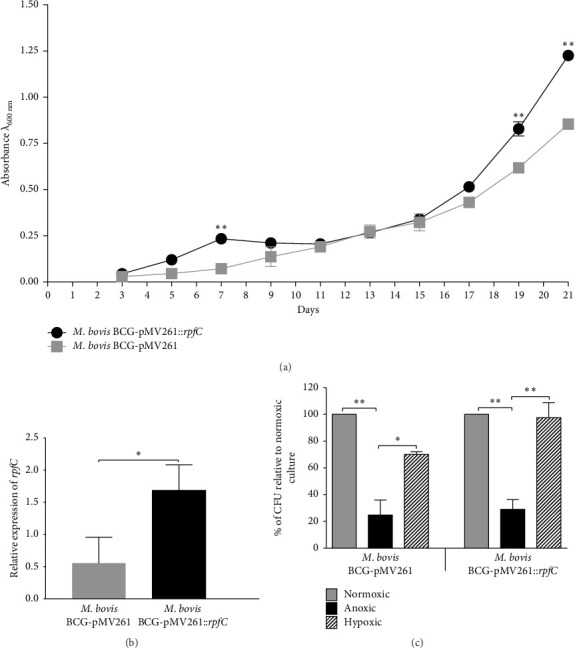
Analysis of *M. bovis* BCG-pMV261::*rpfC* fitness. (a) Growth curve of *M. bovis* BCG-pMV261 and *M. bovis* BCG-pMV261::*rpfC.* (b) Relative expression of *rpfC* in *M. bovis* BCG-pMV261::*rpfC*. (c) Percentage of CFUs of *M. bovis* BCG-pMV261 and *M. bovis* BCG-pMV261::*rpfC* recovered from anoxic and hypoxic cultures. The data are represented as mean ± DE of 2 assays (growth curves) or 3 assays. Statistical significance was determined by a two-way ANOVA test with a Bonferroni correction for growth curves and the Mann–Whitney test for the other essays. ⁣^∗∗^*p* ≤ 0.01; ⁣^∗^*p* ≤ 0.05.

Under normoxic culture conditions, significant differences in the growth of *M. bovis* BCG-pMV261::*rpfC* were observed at the end of the growth curve (days 19 and 21) when a higher growth rate was observed relative to *M. bovis* BCG-pMV261 (Figure 1(a)). The *M. bovis* BCG-pMV261::*rpfC* expresses twofold more *rpfC* (*p* < 0.05) than *M. bovis* BCG-pMV261 (Figure 1(b)).

To evaluate the effect of *rpfC* expression on the viability and growth of *M. bovis* BCG-pMV261 and *M. bovis* BCG-pMV261::*rpfC* cultured in anoxic and hypoxic conditions, the strains were incubated in a normoxic atmosphere for 3 days (to allow recovery of the frozen bacilli) and subsequently incubated in anoxia (0.1% O_2_) for 3 days or hypoxia (5% O_2_) for 5 days, followed by 11 more days under standard culture condition. Both strains showed a notable decrease in CFU recovery after incubation under anoxic conditions. However, the percentage of *M. bovis* BCG-pMV261::*rpfC* CFUs recovered from hypoxic cultures was significantly higher (97%) than that of *M. bovis* BCG-pMV261 hypoxic cultures (70%) (Figure 1(c)). The results suggest that the expression of *rpfC* in *M. bovis* BCG-pMV261::*rpfC* allows the bacteria to remain growing under the hypoxic environment. It is also reflected in the delay of entry to the stationary phase of culture, as is observed in Figure 1(a).

**Figure 2:**
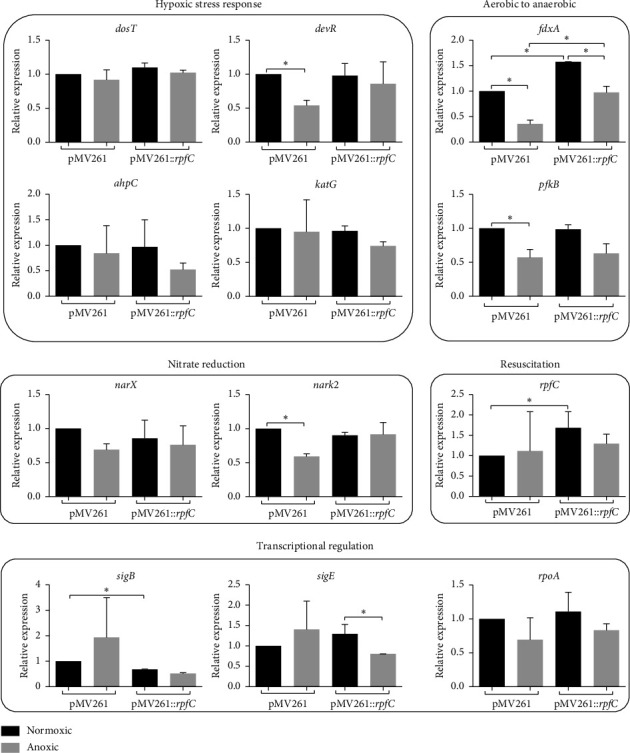
Relative expression of hypoxia-related genes in anoxic cultures. *M. bovis* BCG-pMV261 and *M. bovis* BCG-pMV261::*rpfC* cultures were incubated in anoxic conditions for 3 days. Subsequently, the relative expression of *sigB*, *sigE*, *devR*, *dosT*, *rpoA*, *katG, ahpC*, *fdxA*, *fpkB*, *narX*, *narK2*, and *rpfC* was determined by the 2^−ΔΔCT^ method. Data are represented as mean ± SE of 2 experiments. Statistical significance was determined by a two-way ANOVA test with a Bonferroni correction (post hoc test). ⁣^∗∗^*p* ≤ 0.01; ⁣^∗^*p* ≤ 0.05.

**Figure 3:**
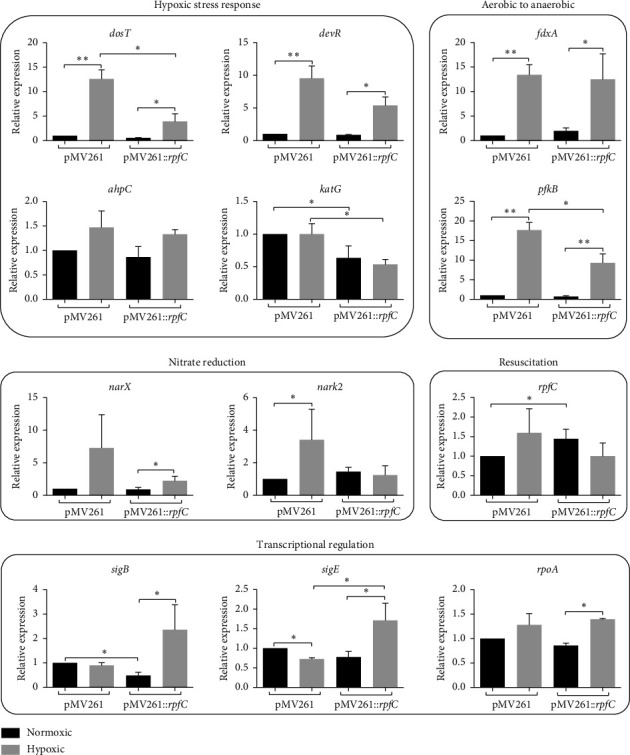
Relative expression of hypoxia-related genes in hypoxic cultures. *M. bovis* BCG-pMV261 and *M. bovis* BCG-pMV261::*rpfC* cultures were incubated in hypoxic conditions for 5 days. Subsequently, the relative expression of *sigB*, *sigE*, *devR*, *dosT*, *rpoA*, *katG, ahpC*, *fdxA*, *fpkB*, *narX*, *narK2*, and *rpfC* was determined by the 2^−ΔΔCT^ method. Data are represented as mean ± SE of 3 experiments. Statistical significance was determined by a two-way ANOVA test with a Bonferroni correction (post hoc test). ⁣^∗∗^*p* ≤ 0.01; ⁣^∗^*p* ≤ 0.05.

To assess the effect of *rpfC* overexpression on the metabolic state of *M. bovis* BCG-pMV261::*rpfC* cultured under normoxic, anoxic, and hypoxic conditions, we evaluate the expression of genes modulated during the hypoxic response and the transition from aerobic to anaerobic metabolism. We chose to analyze the expression of the protein-histidine kinase *dosT* that functions as a hypoxia sensor and the transcriptional switch *devR* as indicative of the activation of the DevR regulon, which in turn upregulate genes necessary for adaptation to hypoxia, such as *fdxA, pfkB, narX*, and *narK2*, which are essential in electron transport and the survival of the bacillus in the transition to anaerobic respiration; the alternative sigma factors *sigB* and *sigE* of response to stress (including hypoxia) and *rpoA* (as indicators of transcriptional activity); the antioxidant response genes *ahpC* and *katG* (which was not differentially regulated in hypoxic stress). The strains were grown for 7 days in a normoxic atmosphere and subsequently incubated in anoxia (< 0.1% O_2_) for three more days (this time was selected because incubating the bacteria for 5 days in anoxia resulted in low-quality RNA and significant heterogeneity among replicates). For hypoxia (5% O_2_), cultures were incubated for 3 days in normoxic conditions and five more days in hypoxia. Figures 2 and 3 show the relative expression of the selected genes in *M. bovis* BCG-pMV261::*rpfC* and *M. bovis* BCG-pMV261 exposed to anoxia and hypoxia.

The overexpression of *rpfC* in *M. bovis* BCG-pMV261::*rpfC* did not significantly affect the expression of most of the genes analyzed, except for *fdxA*, which showed higher expression in comparison with *M. bovis* BCG-pMV261. In anoxic cultures, only a few changes were observed in gene expression in response to the rapid reduction in oxygen tension. Only the expression of *devR, fdxA, pfkB,* and *nark2* in *M. bovis* BCG-pMV261::*rpfC* was lower than in *M. bovis* BCG-pMV261 (Figure 2, *p* < 0.05). Under hypoxic culture, in both strains, the expression of *dosT, devR, fdxA, pfkB*, and *narX* was significantly higher (*p* < 0.5, Figure 3) in comparison with normoxic cultures; however, the expression of the hypoxia-induced genes *dosT, devR,* and *katG* was significantly lower in *M. bovis* BCG-pMV261::*rpfC* (*p* < 0.05) in comparison with *M. bovis* BCG-pMV261. Also, the expression of *pfkB* (of the transition to anaerobic metabolism) and the nitrate reductase *narX* was lower in *M. bovis* BCG-pMV261::*rpfC* (Figure 3). The expression of *ahpC*, *fdxA*, and *rpoA* was similar in both strains; meanwhile, the expression of *sigB* and *sigE* was significantly higher in *M. bovis* BCG-pMV261::*rpfC* (*p* < 0.05) in comparison with *M. bovis* BCG-pMV261. Interestingly, the expression of *rpfC* does not change in *M. bovis* BCG-pMV261::*rpfC* relative to normoxic cultures, where its expression is higher than in *M. bovis* BCG-pMV261.

**Figure 4:**
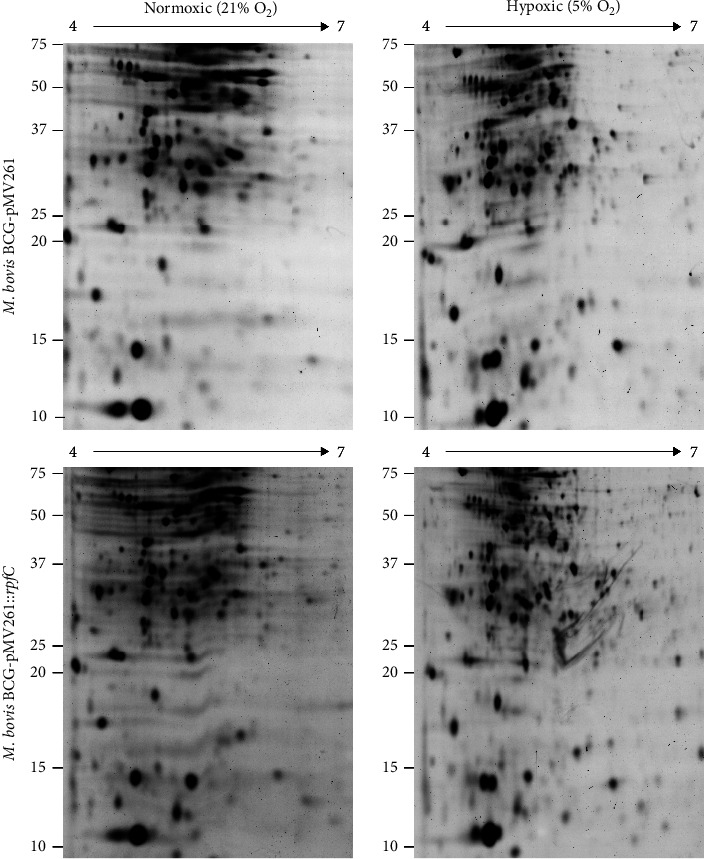
Representative 2-DE proteomes of *M. bovis* BCG-pMV261 and *M. bovis* BCG-pMV261::*rpfC* grown in normoxic and hypoxic conditions. Normoxic cultures were maintained for 8 days at 37°C in an atmosphere of 21% O_2_, while hypoxic cultures were incubated at normoxic conditions for 7 days, followed by 5 days in an atmosphere with 5% O_2_. One hundred μg of cell extract protein was resolved in 7 cm, pH 4-7 strips, and 12.5% SDS-PAGE, and the protein spots were visualized by staining with SYPRO Ruby.

Because the rapid reduction of oxygen concentration in cultures subjected to anoxia does not allow the evaluation of a transition to the quiescent no-replicative state, we only evaluated the differences in the protein profile in the strains grown in hypoxia because, in this condition, we observed significant differences in the expression of several of the hypoxia-response genes, as well as in the viability of the strains studied. We carry out a 2D-PGE analysis to detect differences in the proteome profile of *M. bovis* BCG-pMV261 and *M. bovis* BCG-pMV261::*rpfC* under normoxic and hypoxic culture conditions (Figure 4). In the protein integrity analysis, the expression of HspX was evident in the protein extract of the strains grown in hypoxia (data not shown). Under normoxic culture conditions, the proteomic profile of *M. bovis* BCG-pMV261::*rpfC* presented a higher number of proteins compared to *M. bovis* BCG-pMV261 (an average of 194 and 153 spots, respectively), suggesting that a greater number of cellular processes are active as a consequence of the expression of *rpfC*. A substantial change in the abundance of low molecular weight proteins has been shown to occur in hypoxia. Analysis of differentially expressed proteins revealed the presence of 166 spots consistently detected in normoxic and hypoxic cultures of *M. bovis* BCG-pMV261::*rpfC,* and 28 were differentially expressed in hypoxia. In the proteome of *M. bovis* BCG-pMV261 cultured in hypoxia, the overall number of protein spots detected was 127, of which 11 were only detectable in hypoxia.

## 4. Discussion

Latent tuberculosis (LTB) has received considerable attention in recent years because one-quarter of the world's population is infected with dormant, nonreplicating mycobacteria that persist in the host for years without causing symptoms, thus maintaining a reservoir that makes it difficult to control and eventually eradicate tuberculosis [3, 28]. Understanding mycobacterial reactivation is crucial for the effective control of tuberculosis. During LTB, the mycobacteria survive in an environment with low nutrient availability and a wide range of O_2_ tension, from a hypoxic environment in tissue cells or granulomas to anoxic conditions in necrotic granulomas [11, 29].

*M. tuberculosis* is adapted to an intracellular lifestyle within granulomas in a nonreplicating state induced by low oxygen tension, known as nonreplicating persistence (NRP). Granulomas represent a hypoxic (with a PO_2_ ≤ 10 mmHg) or an anoxic environment in those with central caseation, far below that found in lung tissue, where the PO_2_ of inspired air is 150 mmHg [11, 30]. Hypoxia in granulomas is associated with the transition of replicating *M. tuberculosis* to a dormant, nonreplicating state. Oxygen depletion is a key factor in *M. tuberculosis* dormancy, an NRP characterized by the activation of the three-component regulatory system DosS/DosT/DevR that induces the upregulation genes involved in the fatty acid metabolism and transition to anaerobic respiration, as well as the downregulation of genes of aerobic respiration, replication, transcription, translation, and cell division [31, 32]. Meanwhile, those genes and genes encoding the Rpfs are upregulated during resuscitation [15]. However, the transition from a dormant, nonreplicating state to reactivation remains a puzzle.

In this study, we evaluated the effect of *rpfC* overexpression in *M. bovis* BCG on growth and the expression of DosR-regulated dormancy genes in anoxic and hypoxic cultures. We chose to study RpfC because it is a member of the Rpfs family involved in LTI reactivation, and unlike the other *rpf* genes whose expression changes in some culture phases or stress conditions, *rpfC* is consistently expressed during all *M. tuberculosis* growth phases, under nutrient starvation, in mice models of latent infection, and is induced under gradual hypoxia [19, 24]. Additionally, antibodies against RpfC in the sera from healthy individuals and TB patients and a specific T-cell response in infected children provide indirect evidence of its expression during LTB [26, 33, 34]. Although Rpf proteins are dispensable for the in vitro and in vivo growth of *M. tuberculosis*, it is known that they have a similar function to that described in *M. luteus* [35, 36]. RpfC has peptidoglycan hydrolytic activity and stimulates resuscitation of dormant cells at pM concentration but is not essential for the growth or survival of *M. tuberculosis* and *M. bovis* BCG in liquid culture [18]. Although the mechanism by which Rpfs switch the transition from NRP to active growth is not known, it has been hypothesized that it could be a consequence of stimulating the growth and division of dormant cells as a consequence of the induction of cell wall synthesis or the dispersion of the aggregated bacteria after the hydrolysis of the peptidoglycan. A third hypothesis proposes that the muropeptides resulting from the hydrolysis of the cell function as signaling molecules for dormant cell reactivation [14, 15].

The recombinant *M. bovis* BCG-pMV261::*rpfC* strain was exposed to different oxygen tension conditions, one in which the oxygen concentration quickly decreased to 0.1% (anoxia) and another in which the oxygen decreased to approximately 5% (hypoxia). This model contrasts with Wayne's model, which involves maintaining cultures with moderate agitation in an atmosphere with 5% oxygen that is subsequently decreased to 0.06% [37], and with the rapid anaerobic model that improved oxygen distribution and consumption in the culture until reaching anoxia [38], and other models that combine a hypoxic atmosphere (5% O_2_ and 10% CO_2_) with nutrient starvation and an acidic pH 5.0 [39].

In *M. tuberculosis,* the conditions that inhibit aerobic respiration induce the DosR regulon activation, which is essential for maintaining metabolic and redox homeostasis for bacilli survival in hypoxic and anoxic environments. It has been reported that *dosR* increases its expression up to 80 times during hypoxic stress, and suppressing it results in a hypervirulence phenotype in infected mice and resting macrophages [40]. *devR* is part of the three-component *devR* system that plays an essential role in inducing a genetic program that controls the entry of *M. tuberculosis* into a dormant state [37, 41]. Both *M. bovis* BCG-pMV261 and *M. bovis* BCG-pMV261::*rpfC* present a higher expression of the *dosT* and *devR* genes in response to hypoxia in consistent with various studies demonstrating the higher *dosR* expression in response to hypoxia [42, 43]. However, the expression of both genes was lower in *M. bovis* BCG-pMV261::*rpfC* compared to *M. bovis* BCG-pMV261, probably due to the effect of *rpfC* overexpression under normoxic culture, and the corresponding induction of genes related to replicating bacteria which delay the expression of hypoxia-response genes. Because the mutation of *devR* in *M. tuberculosis* has only a reduced effect on the survival of bacilli in hypoxia, it has been suggested that the activation of the DevR regulon is important as an early response [41]. However, the expression of the DevR regulon is essential for maintaining pH homeostasis and nitrate respiration and is required to recover from dormancy and the resumption of growth [38, 44].

In hypoxic cultures of *M. bovis* BCG-pMV261::*rpfC*, we do not observe significative differences in the expression of *rpfC*, probably because it is already overexpressed in normoxic culture, and continual expression may not be necessary once the protein was synthesized. The metabolic changes that occur during the regulated response induced by the DosR regulon include the expression of genes related to fatty acid metabolism, nitrate respiration, and electron transport because, in hypoxic environments, *M. tuberculosis* obtains energy from alternative carbon sources and the anaerobic reduction of nitrate is essential for the metabolism [37, 41, 45]. The use of anaerobic nitrate reduction for electron transfer is essential for the survival of *M. tuberculosis* in environments with low oxygen tension. The nitrate reductases *narX* and *nirBD* are two of the most critical genes induced by *dosR* during hypoxia, as well as the nitrate transporter NarK2, which is strongly induced during hypoxia to increase nitrate reductase activity [46–48]. However, in *M. bovis* or *M. bovis*, BCG has been reported as a defective induction of *narK2* and *narX* during hypoxia [46, 49]. In concordance with reports, in anoxic cultures, no changes in the expression of *narX* were observed in both strains. However, the expression of *narK2* was significantly lower in *M. bovis* BCG-pMV261 compared to *M. bovis* BCG-pMV261::*rpfC*. In hypoxic cultures, the expression of *narX* and *narK2* was higher (but not significant) in *M. bovis* BCG-pMV261 relative to *M. bovis* BCG-pMV261::*rpfC*. The few changes observed in anoxic cultures must result from the rapid reduction of oxygen tension, which decreases to less than 0.1% in less than an hour. In this condition, we only observed a significantly lower expression of *fdxA*, *narK2* (required for electron transfer in various metabolic reactions), and *devR* in *M. bovis* BCG-pMV261.

In *M. bovis* BCG-pMV261::*rpfC,* the lower expression of the hypoxia-induced genes *dosT*, *katG*, and *pfkB* relative to *M. bovis* BCG-pMV261 is probably due to the *rpfC* overexpression and the effect of the peptidoglycan fragments produced and their effect of growth inducer [50]. In contrast, the sigma factors *sigB* and *sigE,* necessary for stress response, showed at least a twofold higher expression in *M. bovis* BCG-pMV261::*rpfC*. Both *sigE* and *sigB* are induced during in vitro dormancy and are transiently upregulated quickly after re-aeration [15]. These transcription factors could contribute to the positive regulation of other genes that help the bacillus to respond to hypoxia because *sigB* is mainly expressed in the stationary phase, during the resistance to general stress, in response to cell wall damage, and remains induced during the enduring hypoxic response [51]. In contrast, *sigE* is expressed during the exponential growth phase, at the beginning of the stationary phase, under thermal stress conditions, and early during hypoxia [15, 52]. The lower expression of *sigE* observed in *M. bovis* BCG-pMV261 relative to *M. bovis* BCG-pMV261::*rpfC* could be related to a reduced expression of *sigB* because, under physiological conditions, the transcription of *sigB* is dependent on *SigE,* and MprA of the stress-responsive two-component system MprAB directly regulates both transcription factors [53, 54]. On the other hand, in concordance with previous studies [55], the gene that encodes the phosphofructokinase B, a key enzyme of glycolysis, is upregulated under hypoxic conditions as well as *fdxA*, highlighting their importance in adaptation to the reduction of oxygen tension and transition to the hypoxia-induced nonreplicating state. Meanwhile, the *ahpC* gene, which is crucial during hypoxic response and for the persistence of the bacillus [56], showed only a slight induction in response to hypoxia.

We used two-dimensional polyacrylamide gel electrophoresis to compare protein expression in *M. bovis* BCG-pMV261 and *M. bovis* BCG-pMV261::*rpfC* cultured under standard culture conditions and hypoxia. In the proteome of *M. bovis* BCG-pMV261::*rpfC*, we observed more protein spots in normoxic and hypoxic cultures, suggesting that the bacillus remains metabolically active due to the expression of *rpfC*. Both strains showed higher expression of HspX in hypoxia, a protein expressed during hypoxia and potentially involved in latency [57]. Comparative proteomic profiles showed that *M. bovis* BCG-pMV261 expresses approximately 24% fewer proteins in hypoxic culture than in normoxic culture. Meanwhile, in *M. bovis* BCG-pMV261::*rpfC*, the change was minor, and only 15% of the proteins observed in normoxic culture conditions were not detected in hypoxic conditions. The identification of proteins differentially expressed in *M. bovis* BCG-pMV261::*rpfC* under normoxic and hypoxic conditions will allow us to define the changes that induce the expression of *rpfC* in the metabolism of *M. bovis* BCG-pMV261::*rpfC* and understand the pathways by which it promotes the growth and survival of bacteria in hypoxic conditions.

The role of Rpf proteins has been previously studied using knockout strains, adding the proteins to the cultures, or evaluating their expression in different culture phases of or under different stress conditions. This is the first study that evaluates the effect of *rpfC* gene overexpression on the response of the bacteria to hypoxic stress. According to our results, the expression of *rpfC* contributes to maintaining the growth of *M. bovis* BCG-pMV261::*rpfC* in hypoxic cultures, in concordance with the observations of Gupta et al. 2010, which suggests that RpfC may contribute to persistence and stationary-phase adaptation [24]. This result is consistent with in vitro studies that demonstrated that Rpf proteins stimulate the growth of nonreplicating *M. tuberculosis* when added to the culture media and promote the bacteria survival in infected mice [18, 20, 21, 58]. Our results suggest that the overexpression of *rpfC* delays *M. bovis* BCG-pMV261::*rpfC* from entering a nonreplicative stage in hypoxic cultures and induces their growth, as previously described for *M. tuberculosis* in culture or from sputum samples of tuberculosis patients when Rpf proteins are added to the media [12, 20]. Apparently, *rpfC* expression negatively affects the induction of the DosR regulon in response to hypoxia and, consequently, the expression of genes that mediate the transition to anaerobic respiration and the nonreplicative stage. The increased activity of RpfC in hypoxia could be increasing the formation of peptidoglycan fragments that induce the growth of mycobacteria and indirectly stimulate the expression of genes important for maintaining the growth and division of the bacteria. Probably due to this, the expression of several of the hypoxia-response genes is lower in *M. bovis* BCG-pMV261::*rpfC* compared to the strain that does not overexpress *rpfC*. This is also reflected in a minor reduction in protein expression under hypoxic conditions. Due to the expression of resuscitation factors during infection, various authors have recently evaluated their usefulness as subunit vaccines. However, the overexpression of proteins in recombinant vaccines could be a better alternative since, as in this case, the overexpression of *rpfC* favors the growth of the bacillus for a longer time and a better antigenic stimulation. Due to the limitations of the model and the deficiencies that *M. bovis* strains present in the induction of genes related to the respiratory nitrogen metabolites, it is better to use *M. tuberculosis* H37Ra as a model to identify the proteins differentially expressed in response to hypoxia in strains that overexpress *rpfC* or some other Rpfs.

## Figures and Tables

**Table 1:** Primers used for qPCR.

Gene	Primer pair sequence	Tm (°C)	Amplicon length (pb)
sigB	5′-TCG ACA AGA TCA ACG ACC TG-3′	54.6	151
	5′-TGT AAC AGT TCG GCG ATG AC-3′	55.1	

sigE	5′-ACC TGA CCC AGG AGA CCT TT-3′	58	161
	5′-GTC GTA GTC CTC GGG TAA CG-3′	56.8	

rpoA	5′-ATC GAC GAC CTG GAT CTG AC-3′	56.2	141
	5′-CAC CTC GTC GAT GGA CTT CT-3′	56.4	

dosT	5′-CCA TCT TGG CGA TCA CAT C-3′	53.5	150
	5′-CGA TCC CCT CGT AGA CGA AT-3′	56	

devR	5′-CAT CAA GGG AAT GGA GTT GG-3′	53.4	145
	5′-CTG GTC GGT AAG GCC TGA TA-3′	56.1	

katG	5′-GCC GGT CAA GAA GAA GTA CG-3′	55.6	153
	5′-TTG CCC CAA TAG ACC TCA TC-3′	54.4	

ahpC	5′-TTT TGG CCG AAA GAC TTC AC-3′	53.6	160
	5′-TTT TGA GGT CGT TGT GCT GT-3′	55	

fdxA	5′-CTA CAT CAA CCC CGA CGA GT-3′	56.5	143
	5′-GCA GGA CTT GGT GGA AAA AG-3′	54.3	

pfkB	5′-GTC GCT CAT CAA GTC CGT TC-3′	55.9	144
	5′-TAT TGA TCC TGC CCG ACT TC-3′	54.3	

narX	5′-GTG CCG TAT GTG ACG ATG AC-3′	55.9	145
	5′-GGA TGC CGA AAT GAA ACA TC-3′	52.1	

narK2	5′-GCT TGC TGA TGC ACC CTA CT-3′	57.3	140
	5′-TAG GTG GGC AGG TAG TTG CT-3′	58.2	

rpfC	5′-GGG AAC AAC AAA TCG CAG TT-3′	53.9	140
	5′-GCC CAA ATG ATC TCG TTG AT-3′	53.1	

Gapdh	5′-ACC TTA CGG TCG ACT TAT CC-3′	53.6	145
	5′-GAG TCG AAA ATC GAA CTG TG-3′	51.3	

## Data Availability

The data that support the findings of this study are available from the corresponding author upon reasonable request.

## References

[1] Behr M. A., Kaufmann E., Duffin J., Edelstein P. H., Ramakrishnan L. (2021). Latent Tuberculosis: Two Centuries of Confusion. *American Journal of Respiratory and Critical Care Medicine*.

[2] Cardona P. J. (2007). New Insights on the Nature of Latent Tuberculosis Infection and its Treatment. *Inflammation & Allergy Drug Targets*.

[3] Lin P. L., Flynn J. L. (2018). The End of the Binary Era: Revisiting the Spectrum of Tuberculosis. *The Journal of Immunology*.

[4] Barrios-Payan J., Saqui-Salces M., Jeyanathan M. (2012). Extrapulmonary Locations of Mycobacterium Tuberculosis DNA during Latent Infection. *The Journal of Infectious Diseases*.

[5] Neyrolles O., Hernandez-Pando R., Pietri-Rouxel F. (2006). Is Adipose Tissue a Place for *Mycobacterium tuberculosis* Persistence?. *PLoS One*.

[6] Mendez J. E., Zeldon S., Fernando S., Zamora L., José F., Cortés V. (2004). Asdrúbal. Un Acercamiento a la Cinética Del Oxígeno (Parte I). *Revista Costarricense de Cardiología*.

[7] Sullivan M., Galea P., Latif S. (2006). What Is the Appropriate Oxygen Tension for In Vitro Culture?. *MHR: Basic science of reproductive medicine*.

[8] Via L. E., Lin P. L., Ray S. M. (2008). Tuberculous Granulomas Are Hypoxic in Guinea Pigs, Rabbits, and Nonhuman Primates. *Infection and Immunity*.

[9] Carreau A., Hafny‐Rahbi B. E., Matejuk A., Grillon C., Kieda C. (2011). Why Is the Partial Oxygen Pressure of Human Tissues a Crucial Parameter? Small Molecules and Hypoxia. *Journal of Cellular and Molecular Medicine*.

[10] Karakousis P. C., Yoshimatsu T., Lamichhane G. (2004). Dormancy Phenotype Displayed by Extracellular *Mycobacterium tuberculosis* within Artificial Granulomas in Mice. *Journal of Experimental Medicine*.

[11] Kalia N. P., Singh S., Hards K. (2023). *M. tuberculosis* Relies on Trace Oxygen to Maintain Energy Homeostasis and Survive in Hypoxic Environments. *Cell Reports*.

[12] Kana B. D., Gordhan B. G., Downing K. J. (2008). The Resuscitation-Promoting Factors of *Mycobacterium tuberculosis* Are Required for Virulence and Resuscitation From Dormancy but Are Collectively Dispensable for Growth In Vitro. *Molecular Microbiology*.

[13] Shleeva M., Mukamolova G. V., Young M., Williams H. D., Kaprelyants A. S. (2004). Formation of ‘Non-Culturable’ Cells of Mycobacterium Smegmatis in Stationary Phase in Response to Growth under Suboptimal Conditions and Their Rpf-Mediated Resuscitation. *Microbiology*.

[14] Nikitushkin V. D., Demina G. R., Kaprelyants A. S. (2016). Rpf Proteins Are the Factors of Reactivation of the Dormant Forms of Actinobacteria. *Biochemistry*.

[15] Kundu M., Basu J. (2021). Applications of Transcriptomics and Proteomics for Understanding Dormancy and Resuscitation in *Mycobacterium tuberculosis*. *Frontiers in Microbiology*.

[16] Mukamolova G. V., Turapov O. A., Kazarian K. (2002). The Rpf Gene of Micrococcus Luteus Encodes an Essential Secreted Growth Factor. *Molecular Microbiology*.

[17] Gupta R. K., Srivastava R. (2012). Resuscitation Promoting Factors: A Family of Microbial Proteins in Survival and Resuscitation of Dormant Mycobacteria. *Indian Journal of Microbiology*.

[18] Mukamolova G. V., Turapov O. A., Young D. I., Kaprelyants A. S., Kell D. B., Young M. (2002). A Family of Autocrine Growth Factors in *Mycobacterium tuberculosis*. *Molecular Microbiology*.

[19] Russell-Goldman E., Xu J., Wang X., Chan J., Tufariello J. M. (2008). A *Mycobacterium tuberculosis* Rpf Double-Knockout Strain Exhibits Profound Defects in Reactivation from Chronic Tuberculosis and Innate Immunity Phenotypes. *Infection and Immunity*.

[20] Mukamolova G. V., Turapov O., Malkin J., Woltmann G., Barer M. R. (2010). Resuscitation-promoting Factors Reveal an Occult Population of Tubercle Bacilli in Sputum. *American Journal of Respiratory and Critical Care Medicine*.

[21] Downing K. J., Mischenko V. V., Shleeva M. O. (2005). Mutants of *Mycobacterium tuberculosis* Lacking Three of the Five Rpf-Like Genes Are Defective for Growth In Vivo and for Resuscitation In Vitro. *Infection and Immunity*.

[22] Iona E., Pardini M., Mustazzolu A. (2016). *Mycobacterium tuberculosis* Gene Expression at Different Stages of Hypoxia-Induced Dormancy and upon Resuscitation. *Journal of Microbiology*.

[23] Madhur Kalyan S. A., Sharma A., Verma I. (2023). Dormant *Mycobacterium tuberculosis* Reactivates Ex Vivo in Blood from HIV Infected Individuals. *bioRxiv*.

[24] Gupta R. K., Srivastava B. S., Srivastava R. (2010). Comparative Expression Analysis of Rpf-Like Genes of *Mycobacterium tuberculosis* H37Rv under Different Physiological Stress and Growth Conditions. *Microbiology (Reading)*.

[25] Verma A., Kaur M., Singh L. V. (2021). Reactivation of Latent Tuberculosis Through Modulation of Resuscitation Promoting Factors by Diabetes. *Scientific Reports*.

[26] Mattos A. M., Chaves A. S., Franken K. L. (2016). Detection of IgG1 Antibodies against *Mycobacterium tuberculosis* DosR and Rpf Antigens in Tuberculosis Patients Before and After Chemotherapy. *Tuberculosis*.

[27] Castanon-Arreola M., Lopez-Vidal Y., Espitia-Pinzon C., Hernandez-Pando R. (2005). A New Vaccine against Tuberculosis Shows Greater Protection in a Mouse Model with Progressive Pulmonary Tuberculosis. *Tuberculosis*.

[28] Egorova A., Salina E. G., Makarov V. (2021). Targeting Non-Replicating *Mycobacterium tuberculosis* and Latent Infection: Alternatives and Perspectives (Mini-Review). *International Journal of Molecular Sciences*.

[29] Magombedze G., Dowdy D., Mulder N. (2013). Latent Tuberculosis: Models, Computational Efforts and the Pathogen’s Regulatory Mechanisms during Dormancy. *Frontiers in Bioengineering and Biotechnology*.

[30] Aly S., Wagner K., Keller C. (2006). Oxygen Status of Lung Granulomas in Mycobacterium Tuberculosis-Infected Mice. *The Journal of Pathology*.

[31] De Majumdar S., Vashist A., Dhingra S. (2012). Appropriate DevR (DosR)-Mediated Signaling Determines Transcriptional Response, Hypoxic Viability and Virulence of *Mycobacterium tuberculosis*. *PLoS One*.

[32] Honaker R. W., Leistikow R. L., Bartek I. L., Voskuil M. I. (2009). Unique Roles of DosT and DosS in DosR Regulon Induction and *Mycobacterium tuberculosis* Dormancy. *Infection and Immunity*.

[33] Davies A. P., Dhillon A. P., Young M., Henderson B., McHugh T. D., Gillespie S. H. (2008). Resuscitation-Promoting Factors Are Expressed in Mycobacterium Tuberculosis-Infected Human Tissue. *Tuberculosis*.

[34] van Loon W., Gomez M. P., Jobe D. (2020). Use of Resuscitation Promoting Factors to Screen for Tuberculosis Infection in Household-Exposed Children in the Gambia. *BMC Infectious Diseases*.

[35] Tufariello J. M., Jacobs W. R., Chan J. (2004). Individual *Mycobacterium tuberculosis* Resuscitation-Promoting Factor Homologues Are Dispensable for Growth In Vitro and In Vivo. *Infection and Immunity*.

[36] Rosser A., Stover C., Pareek M., Mukamolova G. V. (2017). Resuscitation-promoting Factors Are Important Determinants of the Pathophysiology in *Mycobacterium tuberculosis* Infection. *Critical Reviews in Microbiology*.

[37] Dutta N. K., Karakousis P. C. (2014). Latent Tuberculosis Infection: Myths, Models, and Molecular Mechanisms. *Microbiology and Molecular Biology Reviews*.

[38] Leistikow R. L., Morton R. A., Bartek I. L., Frimpong I., Wagner K., Voskuil M. I. (2010). The *Mycobacterium tuberculosis* DosR Regulon Assists in Metabolic Homeostasis and Enables Rapid Recovery from Nonrespiring Dormancy. *Journal of Bacteriology*.

[39] Deb C., Lee C. M., Dubey V. S. (2009). A Novel In Vitro Multiple-Stress Dormancy Model for *Mycobacterium tuberculosis* Generates a Lipid-Loaded, Drug-Tolerant, Dormant Pathogen. *PLoS One*.

[40] Parish T., Smith D. A., Kendall S., Casali N., Bancroft G. J., Stoker N. G. (2003). Deletion of Two-Component Regulatory Systems Increases the Virulence of *Mycobacterium tuberculosis*. *Infection and Immunity*.

[41] Rustad T. R., Harrell M. I., Liao R., Sherman D. R. (2008). The Enduring Hypoxic Response of *Mycobacterium tuberculosis*. *PLoS One*.

[42] Honaker R. W., Dhiman R. K., Narayanasamy P., Crick D. C., Voskuil M. I. (2010). DosS Responds to a Reduced Electron Transport System to Induce the *Mycobacterium tuberculosis* DosR Regulon. *Journal of Bacteriology*.

[43] Taneja N. K., Dhingra S., Mittal A., Naresh M., Tyagi J. S. (2010). *Mycobacterium tuberculosis* Transcriptional Adaptation, Growth Arrest and Dormancy Phenotype Development Is Triggered by Vitamin C. *PLoS One*.

[44] Reichlen M. J., Leistikow R. L., Scobey M. S., Born S. E. M., Voskuil M. I. (2017). Anaerobic *Mycobacterium tuberculosis* Cell Death Stems from Intracellular Acidification Mitigated by the DosR Regulon. *Journal of Bacteriology*.

[45] Singh P. R., Vijjamarri A. K., Sarkar D. (2020). Metabolic Switching of *Mycobacterium tuberculosis* During Hypoxia Is Controlled by the Virulence Regulator PhoP. *Journal of Bacteriology*.

[46] Sohaskey C. D., Modesti L. (2008). Differences in Nitrate Reduction Between *Mycobacterium tuberculosis* and Mycobacterium Bovis Are Due to Differential Expression of Both narGHJI and narK2. *FEMS Microbiology Letters*.

[47] Philippot L., Hojberg O. (1999). Dissimilatory Nitrate Reductases in Bacteria. *Biochimica et Biophysica Acta (BBA)-Gene Structure and Expression*.

[48] Sohaskey C. D. (2005). Regulation of Nitrate Reductase Activity in *Mycobacterium tuberculosis* by Oxygen and Nitric Oxide. *Microbiology (Reading)*.

[49] Honaker R. W., Stewart A., Schittone S., Izzo A., Klein M. R., Voskuil M. I. (2008). Mycobacterium Bovis BCG Vaccine Strains Lack narK2 and narX Induction and Exhibit Altered Phenotypes during Dormancy. *Infection and Immunity*.

[50] Li X., Ren Q., Sun Z., Wu Y., Pan H. (2024). Resuscitation Promotion Factor: A Pronounced Bacterial Cytokine in Propelling Bacterial Resuscitation. *Microorganisms*.

[51] Lee J. H., Karakousis P. C., Bishai W. R. (2008). Roles of SigB and SigF in the*Mycobacterium tuberculosis*Sigma Factor Network. *Journal of Bacteriology*.

[52] Wayne L. G., Hayes L. G. (1996). An In Vitro Model for Sequential Study of Shiftdown of *Mycobacterium tuberculosis* Through Two Stages of Nonreplicating Persistence. *Infection and Immunity*.

[53] He H., Hovey R., Kane J., Singh V., Zahrt T. C. (2006). MprAB Is a Stress-Responsive Two-Component System that Directly Regulates Expression of Sigma Factors SigB and SigE in *Mycobacterium tuberculosis*. *Journal of Bacteriology*.

[54] Manganelli R., Cioetto-Mazzabo L., Segafreddo G. (2023). SigE: A Master Regulator of *Mycobacterium tuberculosis*. *Frontiers in Microbiology*.

[55] Snasel J., Machova I., Solinova V., Kasicka V., Krecmerova M., Pichova I. (2021). Phosphofructokinases A and B From *Mycobacterium tuberculosis* Display Different Catalytic Properties and Allosteric Regulation. *International Journal of Molecular Sciences*.

[56] Li Z., Kelley C., Collins F., Rouse D., Morris S. (1998). Expression of katG in *Mycobacterium tuberculosis* Is Associated With Its Growth and Persistence in Mice and Guinea Pigs. *Journal of Infectious Diseases*.

[57] Sherman D. R., Voskuil M., Schnappinger D., Liao R., Harrell M. I., Schoolnik G. K. (2001). Regulation of the*Mycobacterium Tuberculosis*hypoxic Response Gene Encoding α-crystallin. *Proceedings of the National Academy of Sciences*.

[58] Biketov S., Potapov V., Ganina E., Downing K., Kana B. D., Kaprelyants A. (2007). The Role of Resuscitation Promoting Factors in Pathogenesis and Reactivation of *Mycobacterium tuberculosis* during Intra-peritoneal Infection in Mice. *BMC Infectious Diseases*.

